# Self-Referential Processing Can Modulate Visual Spatial Attention Deficits in Children With Dyslexia

**DOI:** 10.3389/fpsyg.2019.02270

**Published:** 2019-10-04

**Authors:** Aibao Zhou, Baojun Duan, Menglin Wen, Wenyi Wu, Mei Li, Xiaofeng Ma, Yanggang Tan

**Affiliations:** ^1^School of Psychology, Northwest Normal University, Lanzhou, China; ^2^Key Laboratory of Behavioral and Mental Health, Lanzhou, China; ^3^School of Teacher Education, Hexi University, Zhangye, China; ^4^School of Education, Lanzhou City University, Lanzhou, China

**Keywords:** dyslexia, self-related information, attention, self-reference effect, self-associative learning

## Abstract

Considerable research has shown that children with dyslexia have deficits in visual spatial attention orientation. Additionally, self-referential processing makes self-related information play a unique role in the individual visual spatial attention orientation. However, it is unclear whether such self-referential processing impacts the visual spatial attention orientation of children with dyslexia. Therefore, we manipulated the reference task systematically in the cue-target paradigm and investigated the modulation effect of self-referential processing on visual spatial attention of children with dyslexia. In the self-referential processing condition, we observed that children with dyslexia demonstrated stable cue effects in the visual spatial attention orientation tasks when the Stimulus Onset Asynchronies (SOAs) were set to 100 ms, while other-referential processing weakened the cue effects of the visual spatial attention orientation of children with dyslexia. With cue effect as the index, we also observed that the self-referential processing had a significant larger regulatory effect at the early stage of visual spatial attention orientation, as compared with other-referential processing. These differences have a high-ranked consistency between children with dyslexia and typically developing reader. The results suggested that self-referential processing can regulate the visual spatial attention deficits of children with dyslexia.

## Introduction

Dyslexia is considered to be a special learning disorder that affects the normal reading and spelling ability (Lyon et al., 2003), as it is characterized by difficulties in word decoding and phonological processing abilities (Lyon, 1995). So far, there has been little consensus on the etiology of dyslexia despite the fact that it has been the focus of academic debate (Goswami, 2015; Gori et al., 2016). Numerous studies have shown that phonological awareness may be the core deficit of alphabetic writing in children with dyslexia (Goswami, 2003; Shaywitz et al., 2004; Vellutino et al., 2004; Ziegler and Goswami, 2005; Gabrieli, 2009; Snowling, 2010; Peterson and Pennington, 2012). Many studies in behavioral and cognitive neuroscience have generally acknowledged that deficiencies in the aspects of phonetic perception, manipulation, and recognition may lead to difficulties in grapheme-phoneme correspondence, which hamper the accuracy and fluency of reading (Rumsey et al., 1992; Rae et al., 1998; Temple et al., 2001). In recent years, several studies have indicated that visual spatial attention deficit may be a more basic cognitive factor leading to dyslexia (Brannan and Williams, 1987; Facoetti et al., 2000; Vidyasagar and Pammer, 2010; Zhou et al., 2014; Krause, 2015). Moreover, past studies indicated that self-related information can modulate individual visual spatial attention orientation (Sui et al., 2009a,b; Liu et al., 2016a,b). However, there have been no previous studies regarding whether or how self-related information impact the visual spatial attention of children with dyslexia. As such, this study manipulated the reference task variable systematically, investigating the modulatory role of self-referential processing on visual spatial attention of children with dyslexia.

A number of studies have demonstrated that children with dyslexia have a specific impairment in automatic attentional shifting in response to peripheral visual cues at the short Stimulus Onset Asynchronies (SOAs), reporting no significant cue effect in the cue-target paradigm compared to typically developing readers and adults (Facoetti et al., 2000; Ruffino et al., 2014). Specifically, previous studies have shown that the cueing effect was absent in children with dyslexia when the cue-target interval was shorter (e.g., SOA = 100 ms), but it was present when the cue target interval was longer (e.g., SOA = 250–350 ms) (Facoetti et al., 2005; Ruffino et al., 2014). This attention impairment could be a consequence of a general magnocellular deficit (Facoetti et al., 2003a,b,c). Additionally, other studies have found that the inhibition of return effect in dyslexia was absent in later visual spatial attention processing (Ruffino et al., 2014). Moreover, recent evidence from intervention and longitudinal studies has further demonstrated the causal relationship between visual spatial attention deficits and dyslexia (Facoetti et al., 2003a,b,c; Franceschini et al., 2012). These results, therefore, suggest that the visual spatial attention deficit is an important factor of the etiology of children with dyslexia.

As a regulatory mechanism, self-referential processing plays a unique role in the relationship between self-related information and individual visual spatial attention orientation. For instance, Sui et al. (2009a,b) found that visual spatial attention orientation was regulated by self-related information in the endogenous cueing paradigm, suggesting that self-referential cues are more efficient in capturing visual attention at the early stage of perceptual processing, and shifting voluntary attention at the later stage of perceptual processing. Recently, in a study, Liu et al. (2016a,b) required the participants to complete a cue-target task, where they had to judge the orientation of a lateralized target cued by a central face that dynamically changed its orientation and the results showed that dynamically orienting our own face facilitates the automatic attraction of attention. Moreover, there is considerable evidence indicating that people tend to make much faster and more accurate responses to their own faces compared to the faces of familiar others in visual search and face owner identification tasks (Keenan et al., 1999; Tong and Nakayama, 1999). The self-related information was automatic, unintentional, unconscious, and uncontrolled in the way it affects attentional processes (Alexopoulos et al., 2012). Apparently, these findings provided strong evidence for regulation role of self-referential processing on visual spatial attention orientation.

In recent years, self-associative learning paradigm has provided an effective means to study the regulation of self-related information in the individual cognitive processing. Especially, this paradigm has removed the familiarity effect of own faces and names in self-perception judgment. Using a self-associative learning paradigm, Sui et al. (2012) required participants to associate a neutral geometric shape to three people (e.g., self, friend, and stranger) and then were asked to judge whether subsequent pairs of labels and shapes were matched. They found that participants were quicker and more accurate to match the self-related shape-label, as compared with shape-label matches for other people. Furthermore, shape-label matches to self-related stimuli were relatively unaffected by reductions in contrast. These results suggested that self-related information can regulate basic perception processing and attentional processes. There was a robust advantage to self-related stimuli in all cases, and the different proportion of matched self-pairs did not weaken performance in self-associated matching (Sui et al., 2014). These behavioral effects are sub served by enhanced connectivity between the ventromedial prefrontal cortex (vmPFC) and left posterior superior temporal sulcus (pSTS) (Sui et al., 2013, 2015). Additionally, self-referential processing provides a form of associative “glue” for perception, memory, and decision making, and through this, acts as a central mechanism in information processing. It modulates the mapping between stimuli and perception, memory, and decision making, and also between different stages of information processing (Sui and Humphreys, 2015). These results provided the theoretical basis for regulation of self-referential processing in visual spatial attention orientation. However, to the best of our knowledge, little is known about whether such modulating role of self-referential processing impacts the visual spatial attention orientation of children with dyslexia. Therefore, in the present study, we manipulated the reference task systematically on the cue-target paradigm and examined whether visual spatial attention of children with dyslexia is modulated by different reference tasks.

Mounting evidence has suggested that orthographic depth mediates the role of visual attention in reading (Bavelier et al., 2013; Richlan, 2014). For alphabetic scripts, accurate and rapid attentional shift is needed for segmenting letter strings into its constituent graphemes (Facoetti et al., 2006, 2010). As compared to alphabetic writing systems, visual spatial attention may be particularly important for reading in Chinese because Chinese characters are uniquely distinguished in terms of figure (Yeh and Li, 2002), orthography (Wang, 1973; Hung and Tzeng, 1981), and phonology (Leck et al., 1995; Tan and Perfetti, 1997). Moreover, Chinese characters are composed of multiple strokes or radicals within a two-dimensional space. Therefore, accurate visual spatial attention is needed to process character configuration accurately and efficiently. In addition, there are no word boundaries for Chinese texts and effective attention may help Chinese readers identify words in a sentence with an independent visual unit (Liu et al., 2015, 2016a,b). Taken together, previous studies have provided strong evidence that visual spatial attention should play an important role in Chinese reading at both the character and text levels.

There are few empirical studies conducted on visual spatial attention orientation in Chinese children with dyslexia. Recently, only a study using the cue-target paradigm indicated that covert attentional shifting was selectively impaired in Chinese children with dyslexia (Ding et al., 2016). Thus, it revealed that visual spatial attention impairment is consistent across different writing systems. Unfortunately, this study investigated the later stage of visual spatial attention by measuring inhibition of return (IOR), which did not involve the facilitation effect in the earlier processing of visual spatial attention in Chinese children with dyslexia.

Based on the previous studies, the primary aim of the present study was to investigate the modulatory role of earlier stage of visual spatial attention in Chinese children with dyslexia. To this end, we manipulated reference tasks in the cue-target paradigm. That is, in the condition of self-referential processing, the subjects were asked to be self-centered in order to determine whether the target stimulus was present. Meanwhile, in the other-referential processing condition, participants were asked to be other-centered in order to determine whether the target stimulus was present. Therefore, we hypothesized that self-referential processing could regulate the early stage of visual spatial attention processing in Chinese children with dyslexia, while other-referential processing weakened the regulation of early deficits in visual spatial attention processing.

## Materials and Methods

### Participants

Twelve children with dyslexia (male: 11; *M*_age_ = 116.50 months, SD_age_ = 14.48 months) and 13 age-matched typically developing readers (male: 7; *M*_age_ = 113.77 months, SD_age_ = 11.26 months) participated in this study. These children were selected from 442 third-grade to fifth-grade students in a local elementary school. The participants were selected using a standardized test of nonverbal intelligence, the Raven’s Standard Progressive Matrices (Raven et al., 1998), with local norms established by Zhang and Wang (1985), and a Character Recognition Measure and Assessment Scale for Primary School Children (Wang and Tao, 1993), which has been widely used for screening Mandarin-speaking Chinese children for dyslexia (Shu et al., 2006). On this standardized battery of Chinese character recognition measure and assessment scale, participants were required to write down a compound word based on a constituent morpheme provided on the sheet. The children with dyslexia demonstrated reading achievement scores of at least 1.5 years below their corresponding age. The age-matched typically developing readers came from the same schools and were no more than 1.5 years below on the same measure. All of them were native Chinese speakers, with normal or corrected-to-normal vision. No participant had a co-morbid diagnosis of ADHD, as established by the Conners Child Behavior Rating Scale. The study was approved by the local ethical committee of Northwest Normal University and written informed consent was obtained from the parent or teacher of each participant.

Upon examination, there was a significant difference between dyslexic children and typically developing reader in character recognition measure, *t* (20) = −5.76, *p* < 0.01, Cohen’s *d* = 2.45, indicating lower scores in dyslexic children than typically developing reader. However, no significant difference was found in relation to age, *t* (20) = 0.61, *p* > 0.05, Cohen’s *d* = 0.26, and nonverbal intelligence test, *t* (20) = −1.73, *p* > 0.05, Cohen’s *d* = 0.73.

### Stimuli and Apparatus

The experiment had two phases. In the first phase, we used an adapted version of Sui et al.’s (2012) self-associative learning paradigm with neutral geometric shapes. One of two geometric shapes (circle or square, each 3.8° × 3.8°) was presented above a black fixation cross. The fixation cross (0.8° × 0.8°) was displayed in the center of the screen. The distance between the center of the geometric shape or the word and the fixation cross was 3.5°. All stimuli were shown on a gray background. The two geometric shapes were associated with two labels (self, or a stranger named Wang Hua, each 3.1° × 3.1/6.5°) and counterbalanced across participants. The Chinese word “我” (which means self) or “王华” (the name of a stranger) was displayed below the fixation cross. The distance between the center of the shape or the word and the fixation cross was 3.5°. The participants were required to judge whether briefly presented shape-label pairings were correct or incorrect (e.g., “Does the circle represent yourself?” and “The circle represents a stranger, Wang Hua?”).

After the self-associative learning phase, we adopted the paradigm used by Ruffino et al. (2014) to assess visual spatial attention orientation of participants. In his paradigm, one of two geometric shapes (circle or square, each 2.5° × 2.5°) was presented peripherally to the left and to the right of the fixation point (1° visual angle). The cue was displayed as an arrow (1.5° visual angle) above one of the geometric shapes. The target was a dot (0.5° visual angle), presented after one of the two cue-target stimuli onset asynchronies in one of the possible locations. E-Prime software (version 2.0) was used to present the stimuli and record responses and the experiment was displayed on a 17-inch monitor (1,024 × 768 at 60 Hz).

### Experimental Procedure

All participants were tested in a quiet room and completed the experimental tasks alone. All experimental stimuli were presented 40 cm from the computer screen on a gray background. The experimental procedure was divided into two stages.

**Figure 1:**
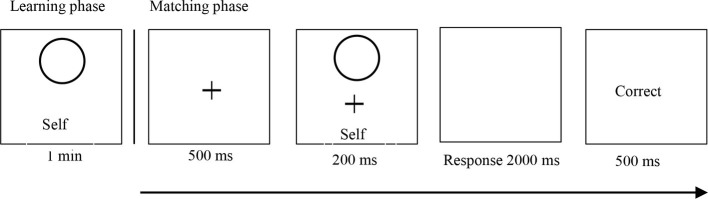
Schematic representation of the label-matching task (Payne et al., 2016). In the self-associative learning phase, one of the two geometric shapes and identity labels (self and other) are presented on screen for 1 min. In the matching phase, each trial starts with a fixation cross (500 ms), followed by a shapes–label pair (200 ms), after which the participant has 2,000 ms to respond. Following the response, visual feedback is presented for 500 ms before the start of the next trial.

In the self-associative learning phase, participants were trained to associate self and Wang Hua with the two types of geometric shape (circle and square). Participants were told which shape was associated with the self or Wang Hua. For example, a participant was told, “you are a circle, and Wang Hua (a stranger for the participant) is represented by a square.” The assignment of shape with the self was counterbalanced across participants. The procedure is illustrated in Figure 1. Participants had 60 s to learn the shape-label pairings before starting the matching task. After this, each trial began with a fixation cross for 500 ms. The shape-label pairing either corresponded with a pairing seen by participants during matching phase or was a mismatch trial. Participants responded by either pressing the P key using their left hand or pressing the Q key using their right hand. Participants were expected to judge whether the shape was correctly assigned to the person by pressing one of the two response buttons as quickly and accurately as possible within this timeframe. The automatic feedback (“correct” or “incorrect”) was then presented following each trial for the participants, lasting 500 ms. Each participant performed two blocks of 120 trials following 12 practice trials, where self, Wang Hua, and re-paired stimuli occurred equally often in a random order. Thus, there were 60 trials in each condition (self-matched, self-nonmatching, other-matched, and other-nonmatching). Participants were informed of their overall accuracy at the end of each block.

**Figure 2:**
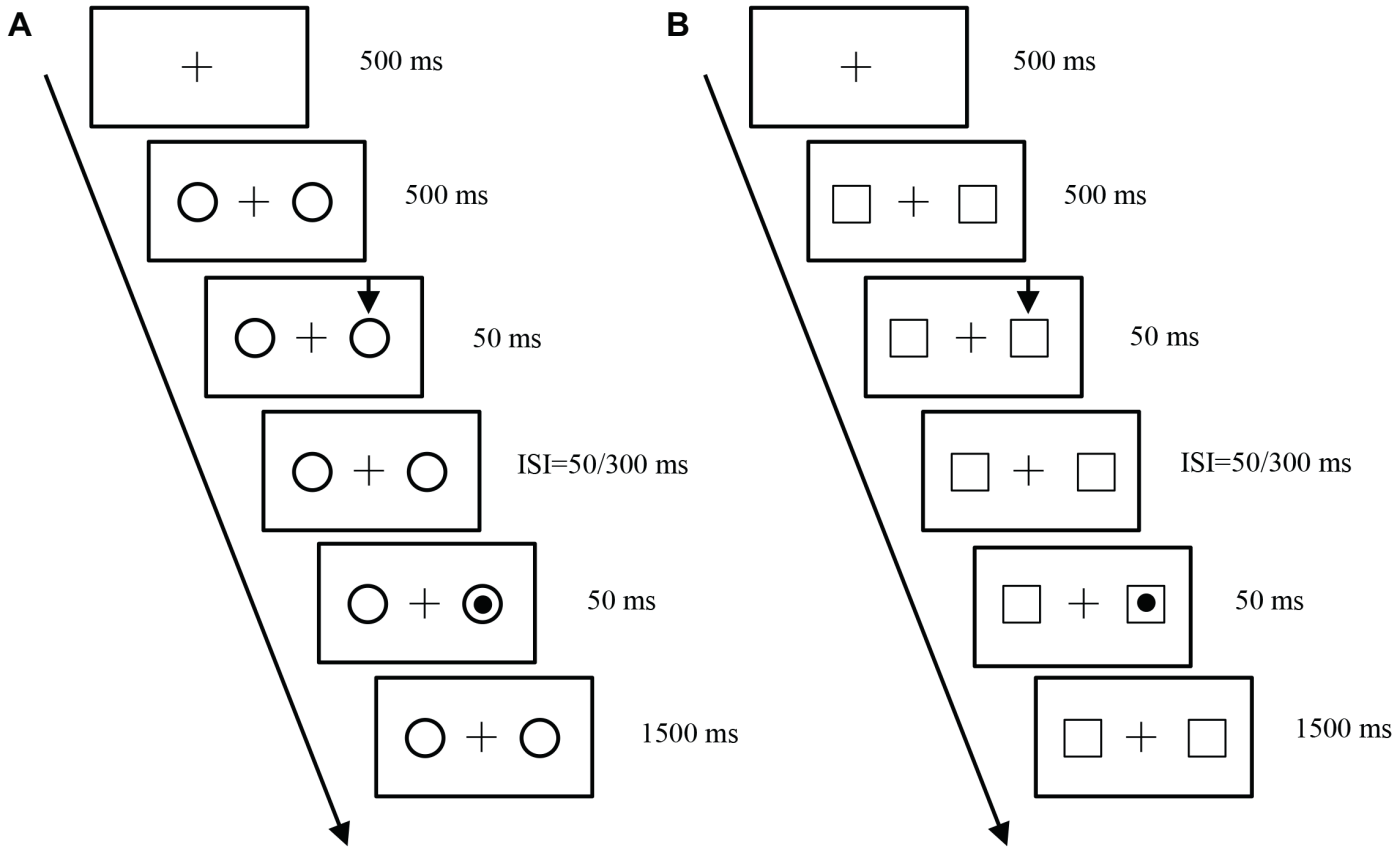
Schematic representation of the display used in the visual spatial attention (Ruffino et al., 2014). **(A)** Schematic description of a trial for measuring the visual spatial attention of self-reference processing condition. Each trial started the onset with the fixation point; two circles (the circles represented “self”) were presented peripherally to the left and to the right of the fixation point. The cue was displayed as an arrow above one of the circles. The dot target was presented after one of two cue-target inter-stimulus interval (ISI, 50 or 300 ms) in one of the two possible locations. Observers were instructed to press the spacebar button to react as quickly as possible to the onset of the target. **(B)** Schematic description of a trial for measuring the visual spatial attention of other-reference processing condition. The squares represented “other.” The experimental procedures and tasks were same as self-reference processing condition.

In cue-target judgment phase, the stimuli were same as those in the self-associative learning phase. Participants were seated in front of a computer screen and their head was positioned on a chinrest so that the eye-screen distance was 40 cm. Participants were instructed to keep their eyes on the fixation point throughout the duration of the trial. The experimental procedure was divided into self-reference processing condition and other-reference processing condition, respectively. In self-reference processing condition (see Figure 2A), each trial started the onset with the fixation point, where one of two types of geometric shapes was displayed peripherally to either side (one to the left and one to the right of the fixation point). The circles represented “self.” The visual cue was shown 500 ms later, and it consisted of an arrow displayed for 50 ms above one of the circles. On each response trial, a dot target was presented after one of two cue-target stimulus onset asynchronies (SOA, 100 or 350 ms) in one of the two possible locations. In other-reference processing condition (see Figure 2B), the squares represented “other” and the experimental procedures and tasks were similar to self-reference processing condition. The two types of geometric shapes were counterbalanced across participants. The probability that the cue was presented in the target location was about 80% (i.e., the cue location was predictive of target location), and the probability of an invalid cue trial was about 10% (i.e., the cue location was not predictive of target location). In contrast, in catch trials, the target was not presented and participants did not have to respond. Catch trials were intermixed with response trials and were about 10% of the trails. The experimental session consisted of 128 trials divided into two blocks of 64 trials each, including 40 valid trials (20 targets in the right visual field and 20 in the left visual field, 10 for each SOA), 12 invalid trials (6 targets in the right visual field and 6 in the left visual field, 3 for each SOA), and 12 catch trials (6 for each SOA). Participants were instructed to react as quickly as possible to the onset of the target by pressing the spacebar on the computer keyboard. Both simple RTs and error rates were recorded by the computer. The maximum time response allowed was 1,500 ms, the inter-trial interval was 1,000 ms, after that time the trial started automatically. The participants underwent eight practice trials and were given formal experiments.

## Results

### The Shape-Label Matching Phase

**Figure 3:**
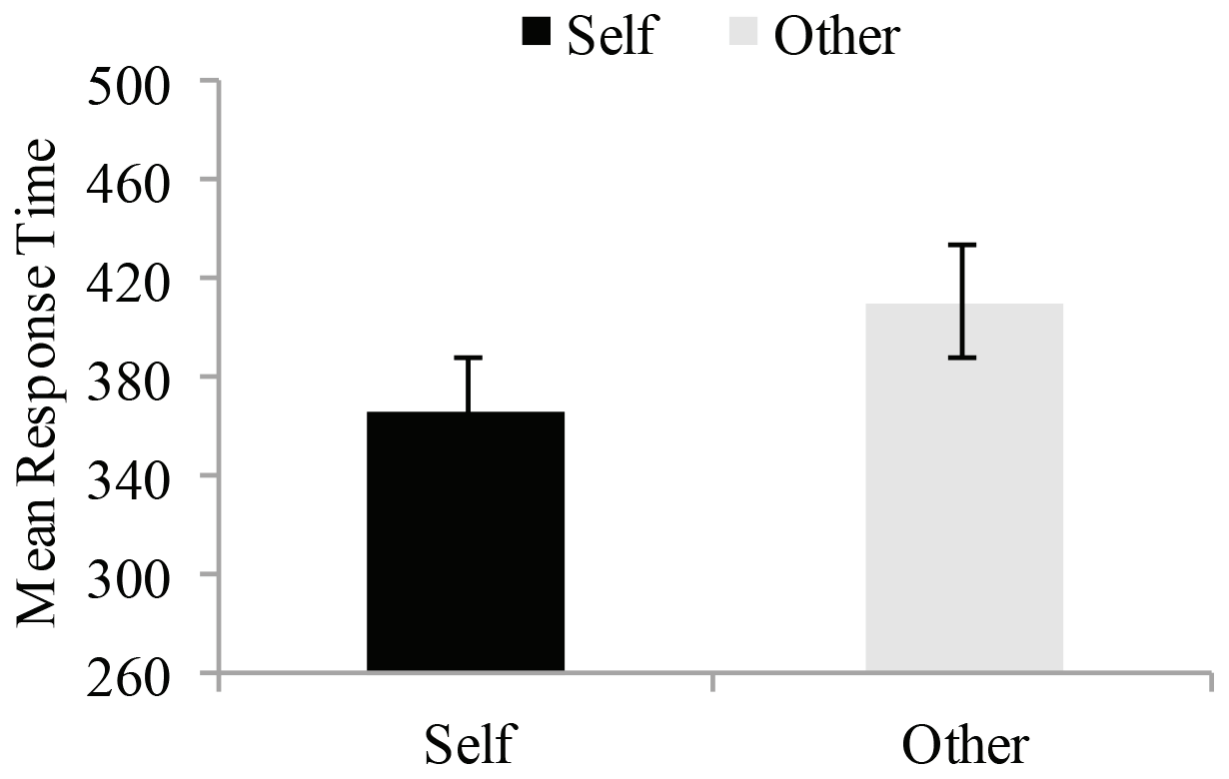
Mean response time in the self-perception matching and other-perception matching tasks. Mean response time are plotted as a function of different shape-label matching tasks for each of the participants. The data were collected using standard methods for the lab (Sui et al., 2012; Yin et al., 2019). Error bars denote 1SEM calculated across subjects for each condition.

Outliers were excluded from the datasets before the analyses were carried out. In the present experiment, the RTs less than 200 ms and greater than 1,000 ms were not considered in our analyses (Sui et al., 2012; Payne et al., 2016). These excluded trials accounted for 6.55 and 5.50% of the trials tested in the children with dyslexia and typically developing readers, respectively. Furthermore, during the experimental process, two subjects did not complete all the experiment tasks. Due to the RTs more than 2.5 SD above the mean RT (Facoetti et al., 2003a,b,c, 2005), the mean RT of one subject was regarded as an outlier. As such, the residual experimental data from 22 subjects were analyzed (see Table 1). On the basis of prior research (Yin et al., 2019), only correct responses with RTs were analyzed with a mixed ANOVA in which the within-subject factor was reference mode (self-reference vs. other-reference) and the between-subject factor was group (typically developing reader vs. dyslexia). A significant main effect was found for reference mode *F* (1, 16) = 14.09, *p* < 0.05, *η*^2^ = 0.47, the correct responses with RTs were faster in the self-reference matching condition than other-reference matching condition. The main effect of group was not significant *F* (1, 16) = 0.11, *p* > 0.05, *η*^2^ = 0.001. The reference mode × group interaction was not significant *F* (1, 16) = 2.41, *p* > 0.05, *η*^2^ = 0.13 (see Figure 3).

**Table 1:** Summary mean (SD) reaction times, accuracies and *d*′ value for self-associative learning phase.

Group	Reference mode	Mean RT	*d*′
DD (*N* = 11)	Self	373.53 (21.99)	3.19 (1.85)
	Other	399.71 (24.31)	3.00 (1.13)
TD (*N* = 11)	Self	358.56 (24.99)	3.41 (1.32)
	Other	421.66 (27.63)	3.33 (1.17)

*DD, children with dyslexia; TD, typically developing reader; RT, response time*.

**Figure 4:**
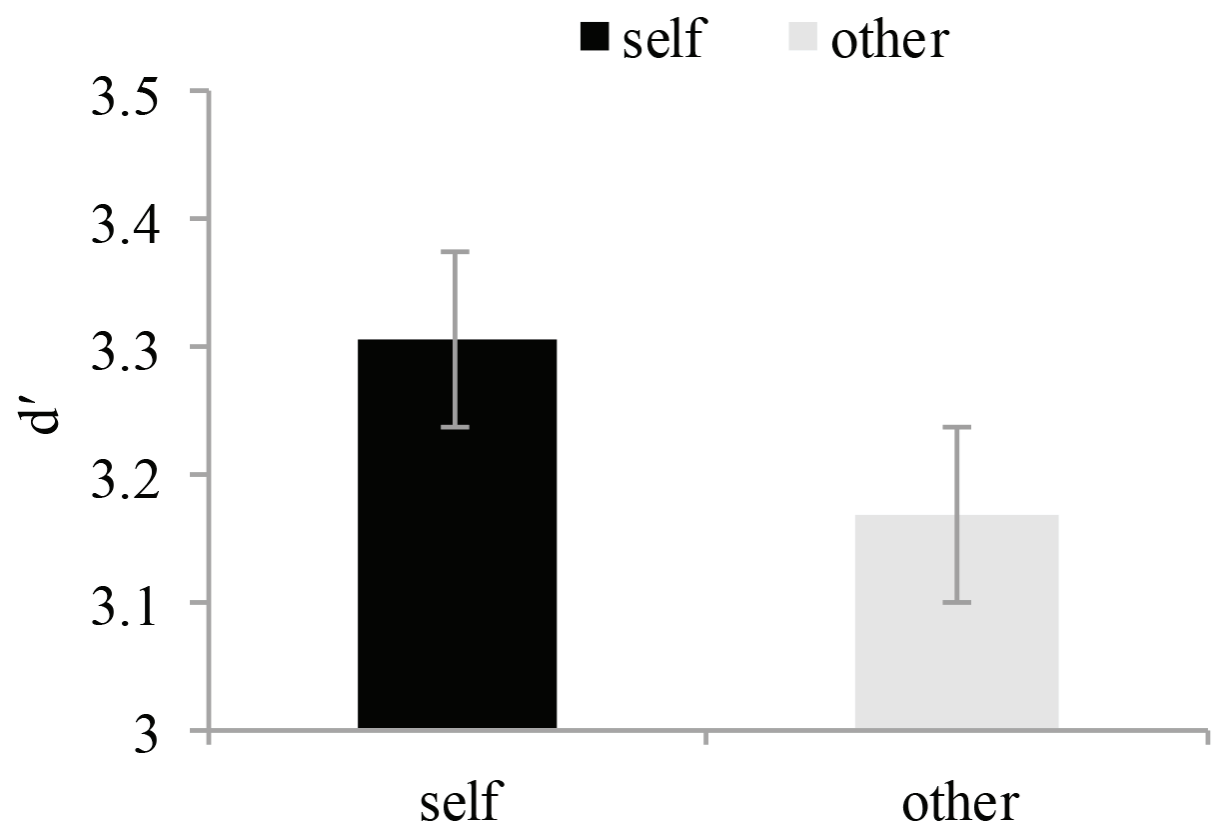
The *d*′ value of the self-perception matching and other-perception matching conditions. The *d*′ value is plotted as a function of different shape-label matching conditions. Error bars denote 1SEM calculated across subjects for each condition.

To access the self-prioritization effect, we analyzed *d*′ value using a signal detection approach. The *d*′ value, reflecting perceptual sensitivity to each label-matching performance, was calculated by combining performance in each label-matching condition across both match and mismatch trials. We used a 2 × 2 mixed ANOVA on the *d*′ score, the within-subject factor was reference mode (self-reference vs. other-reference), and the between-subject factor was group (typically developing reader vs. dyslexia). There was a significant effect of reference mode *F* (1, 20) = 4.46, *p* < 0.05, *η*^2^ = 0.18, and the *d*′ score was larger for the self-reference matching condition than the other-reference matching condition. On the other hand, there was no significant effect of group for the *d*′ score, *F* (1, 20) = 0.15, *p* > 0.05, *η*^2^ = 0.007. In addition, the interaction between the reference mode and the group was not significant (see Figure 4).

### Cued-Target Detection Task

In order to systematically investigate the effects of reference task on the visual spatial attention of typically developing reader and children with dyslexia, a repeated measures ANOVA with reference mode (self-reference vs. other reference), SOA (100 vs. 350 ms) and target condition (valid vs. invalid) as within-subject variables and the group (dyslexia vs. typically developing reader) as between-subject variable revealed that the main effects of reference mode was significant, *F* (1, 16) = 6.19, *p* < 0.05, *η*^2^ = 0.28, reflecting faster RTs for the other reference condition than the self-reference condition. The main effect of target condition was significant, *F* (1, 16) = 13.15, *p* < 0.05, *η*^2^ = 0.45, reflecting faster RTs for the valid condition than the invalid condition. The main effect of SOA and group were both not significant, *F* (1, 16) = 0.13, *p* > 0.05, *η*^2^ = 0.008, *F* (1, 16) = 0.09, *p* > 0.05, *η*^2^ = 0.006. More importantly, the interaction between reference mode, SOA, and target condition was significant, *F* (1, 16) = 7.79, *p* < 0.05, *η*^2^ = 0.33. However, this effect did not interact with group, *F* (1, 16) = 0.43, *p* > 0.05, *η*^2^ = 0.03, showing that the regulation effects of reference mode on SOA and target condition are independent across group.

**Figure 5:**
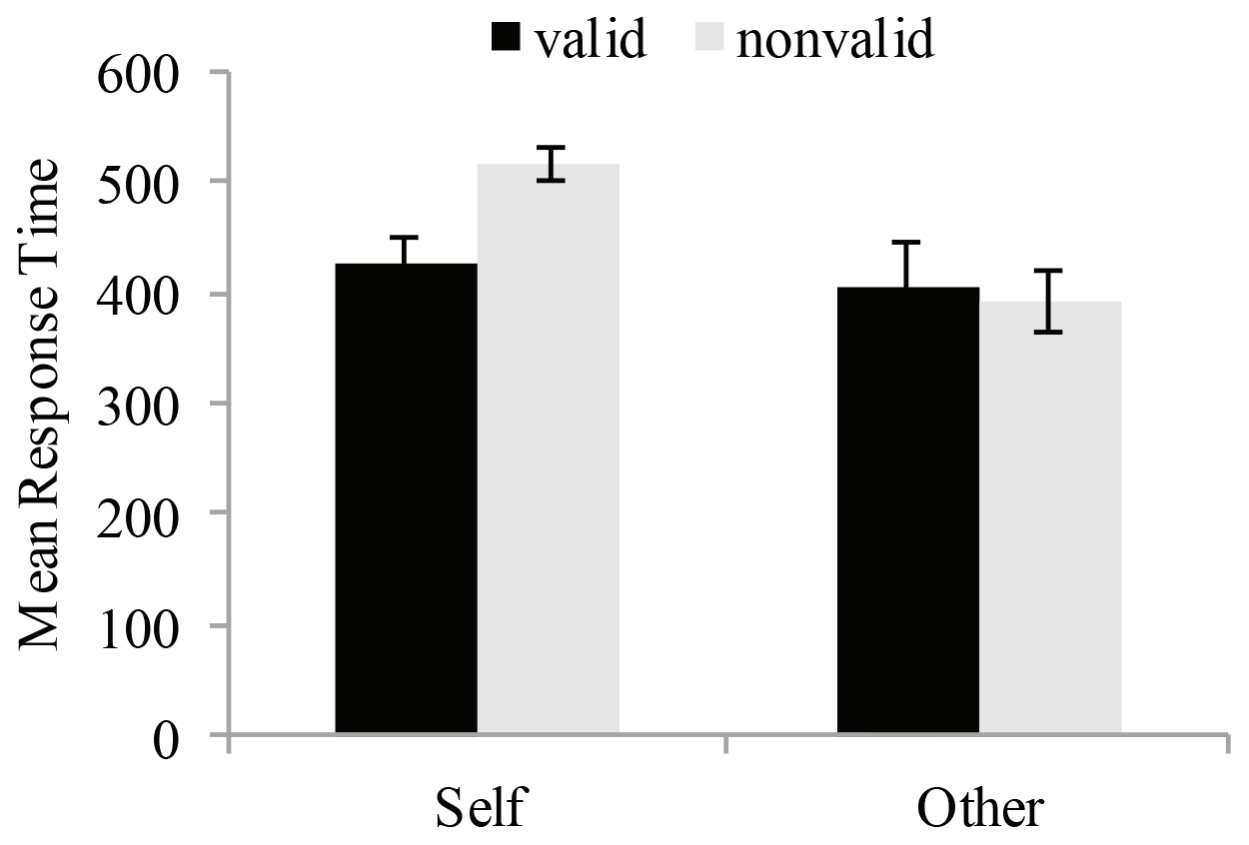
Results of the 100 ms SOA condition. Data are plotted as a function of different reference mode and cue mode conditions for children with dyslexia and typically developing readers. Error bars denote 1SEM calculated across subjects for each condition.

**Figure 6:**
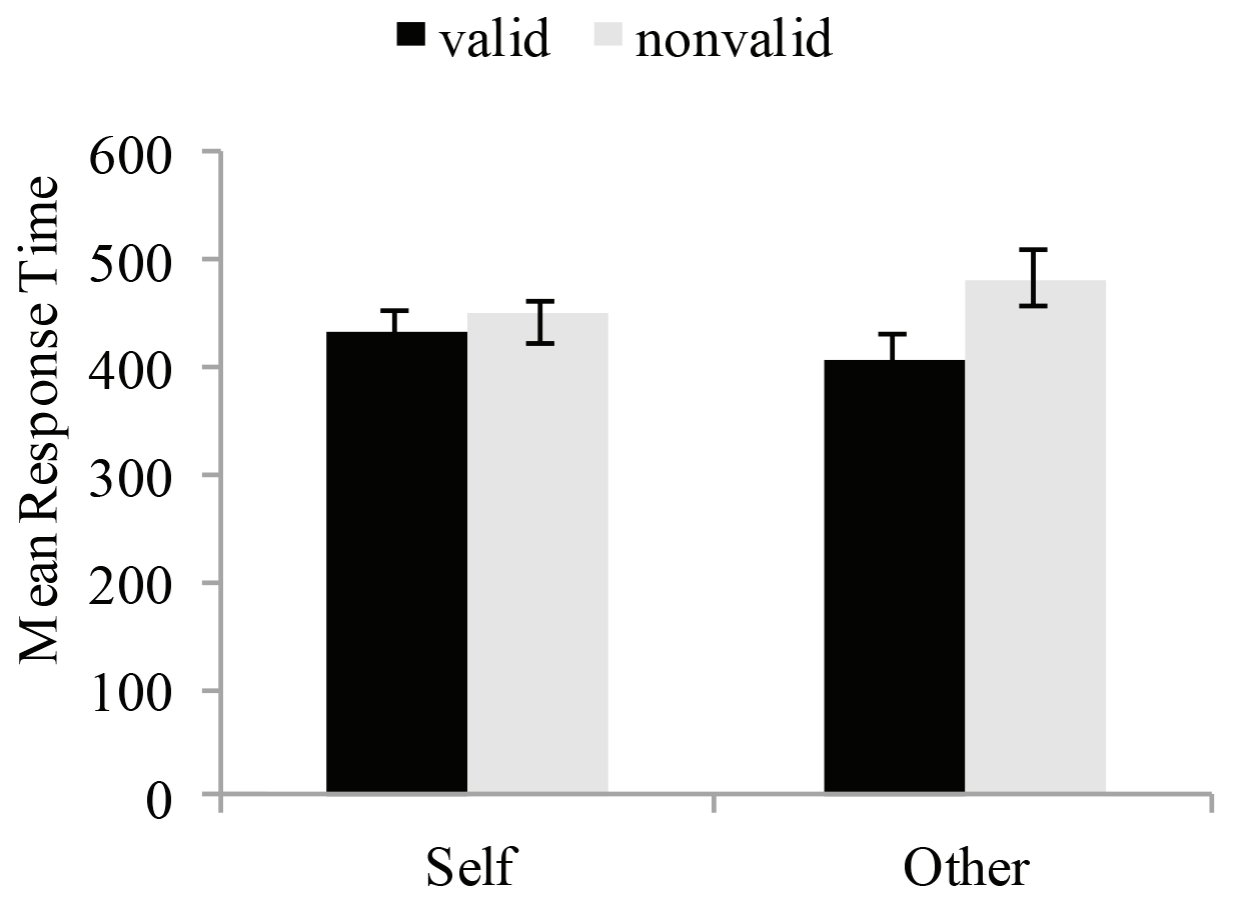
Results of the 350 ms SOA condition. Data are plotted as a function of different reference mode and cue mode conditions for children with dyslexia and typically developing readers. Error bars denote 1SEM calculated across subjects for each condition.

In order to establish whether or not there would be a difference on the cue effect of the two different SOAs of visual spatial attention, we adopted repeated measures ANOVA with reference mode (self-reference vs. other reference) and target condition (valid vs. invalid) as within-subject variables on the mean RTs (see Table 2). At the 100 ms SOA condition, the reference mode showed a significant effect on the mean RTs, *F* (1, 19) = 8.37, *p* < 0.05, *η*^2^ = 0.31, indicating that the mean RTs was significantly higher in self-referential processing than other-referential processing. Also, the main effect of target condition was significant, *F* (1, 19) = 5.48, *p* < 0.05, *η*^2^ = 0.22, reflecting faster RTs for the 100 ms than the 350 ms condition. More importantly, the interaction between the reference mode and the target condition was significant, *F* (1, 19) = 7.48, *p* < 0.05, *η*^2^ = 0.28. In the self-referential processing, valid cues had faster RTs than invalid cues, *F* (1, 19) = 9.91, *p* < 0.05, whereas in the other-referential processing, valid cues and invalid cues did not differ, *F* (1, 19) = 0.54, *p* > 0.05 (see Figure 5). On the other hand, at the 350 ms SOA condition, there was a significant main effect of target condition, *F* (1, 19) = 6.18, *p* < 0.05, *η*^2^ = 0.25, reflecting faster RTs for the valid condition than the invalid condition. The main effect of reference mode was not significant, *F* (1, 19) = 0.02, *p* > 0.05, *η*^2^ = 0.001. Moreover, the interaction between target condition and reference mode was not significant, *F* (1, 19) = 3.27, *p* > 0.05, *η*^2^ = 0.15 (see Figure 6).

**Table 2:** Summary mean (SD) reaction times for the 100 ms SOA condition and the 350 ms SOA condition.

SOAs	Self		Other	
	Valid	Nonvalid	Valid	Nonvalid
100 ms	424.09 (24.01)	515.83 (37.43)	406.52 (16.26)	391.00 (27.92)
350 ms	433.91 (16.42)	449.86 (25.45)	406.34 (13.41)	482.60 (27.00)

**Figure 7:**
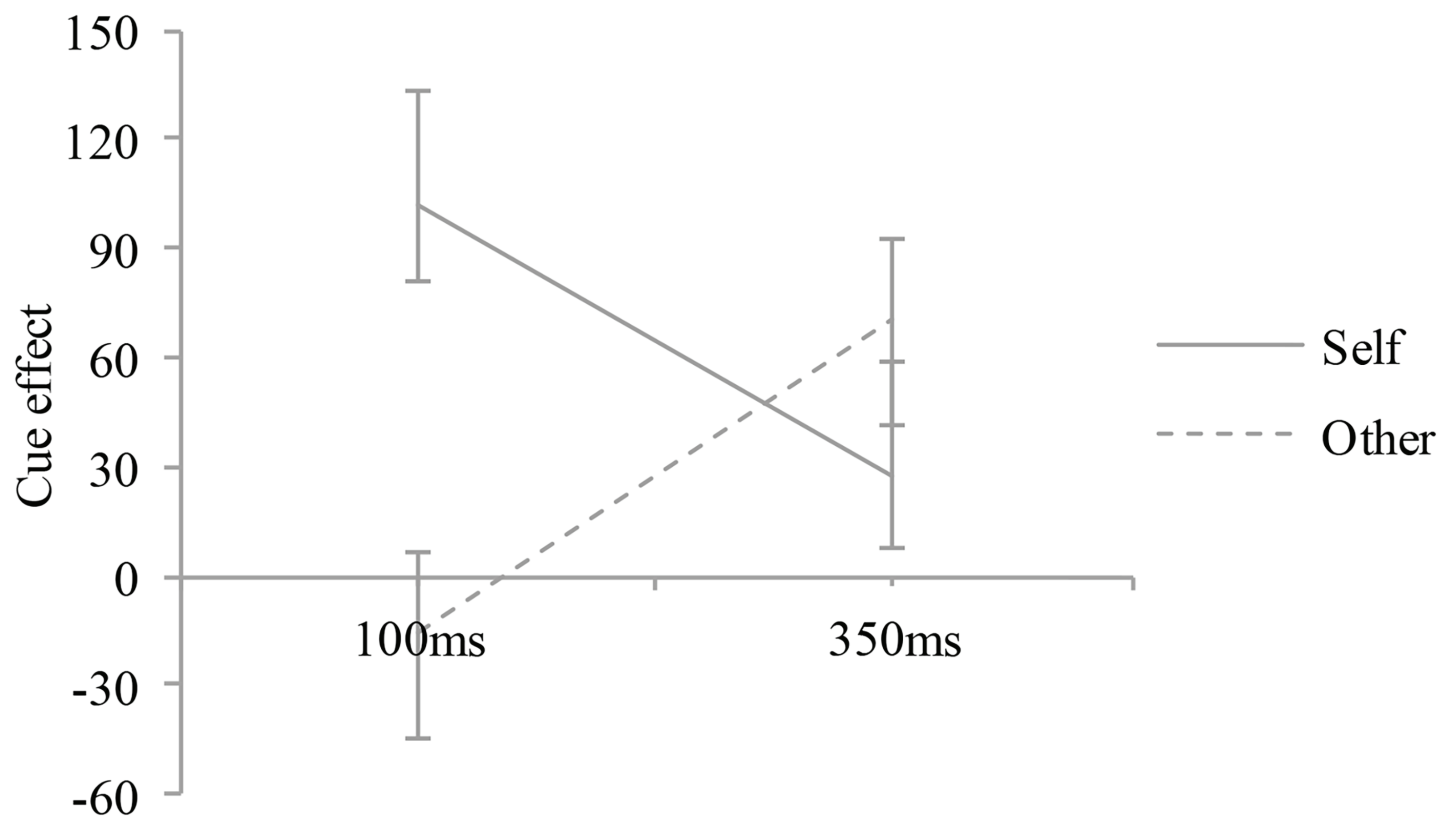
Effect of the reference mode on the early stage of visual spatial attention. The cue effect is plotted as a function of different reference mode and SOA conditions for children with dyslexia and typically developing readers. Error bars denote 1SEM calculated across subjects for each condition.

To further study the regulatory role of different reference tasks on the visual spatial attention orientation, we analyzed the results of two different SOAs of visual spatial attention comprehensively. The cue effect (i.e., invalid – valid cue conditions) was analyzed by means of a mixed ANOVA in which the two within-subject factors were reference mode (self-reference vs. other reference) and SOA (100 vs. 350 ms). The between-subject factors were group (dyslexia vs. typically developing reader). The interaction between the reference mode and the SOA was significant, *F* (1, 16) = 7.79, *p* < 0.05, *η*^2^ = 0.33, indicating that cue effect at the two different SOAs varied across reference mode. Specifically, at the 100 ms SOA, the reference mode showed a significant effect on the cue effect, *F* (1, 16) = 7.83, *p* < 0.05, indicating that the cue effect was significantly higher in self-referential processing than other-referential processing. In contrast, at the 350 ms SOA, the reference mode showed no significant effect on the cue effect, *F* (1, 16) = 1.78, *p* > 0.05. The reference mode × SOA × group interaction was not significant, *F* (1, 16) = 0.43, *p* > 0.05, *η*^2^ = 0.03, indicating that the interaction between the reference mode and the SOA was very consistent across children with dyslexia and typically developing readers (see Figure 7). Based on the above analysis, we found the modulation effect of the visual spatial attention orientation of children with dyslexia under the 100 ms SOA condition, as compared with the 350 ms SOA condition.

## Discussion

Based on self-associative learning task, the present study manipulated the reference task in the cue-target paradigm systematically, investigating the modulatory role of self-referential processing and other-referential processing on visual spatial attention of children with dyslexia. Firstly, we conducted a replicate experiment to ensure the subjects responded faster under self-associative condition, as compared to other association condition. The result of the present study was consistent with previous research and demonstrated a self-association advantage effect on simple perceptual matching tasks (Sui et al., 2012; Yin et al., 2019). These results suggested that the participants established a temporary connection between label matching and social significance in the perceptual matching task.

Our main aim in this study was to explore the regulation of self-referential processing on visual spatial attention of children with dyslexia. Consistent with our hypothesis, we found that subjects demonstrated a stable cue effect at the early stage of visual spatial attention orientation by manipulating the reference mode variable. Specifically, at 100 ms SOA condition, the result showed that mean RTs was significantly faster in valid cues than invalid cues only in the self-referential processing condition, but failed to observe a significant difference between valid cues and invalid cues in the other-referential processing. These findings suggest that children with dyslexia showed significant facilitation effect of early stage of visual spatial attention under the self-referential processing condition. There are two main possible reasons why the self-referential processing may induce facilitation effect of early stage of visual spatial attention for children with dyslexia. One possible reason for this finding is that previous evidence has shown that the self-referential processing enhanced the task performance of self-related information within perception (Sui et al., 2012), attention (Sui et al., 2009a,b; Sui and Humphreys, 2015), and within memory (Rogers et al., 1977; Symons and Johnson, 1997). Rogers et al. (1977) suggested that self-reference processing is a rich and powerful encoding process due to the effect of self-schema on memory encoding and retrieval, which result in the self-reference effect. Numerous studies have shown that the self-related information processing is characterized by faster responses and better memory performance (Bundesen et al., 1997; Keenan et al., 1999; Tong and Nakayama, 1999; Sui et al., 2009a,b; Keyes and Brady, 2010), compared to others or semantic level (Rogers et al., 1977; Symons and Johnson, 1997). These self-reference effect accounts for distinguishing stimuli related to one’s own self from those that are not relevant to one’s own concerns (Northoff et al., 2006). Additionally, self-related integrative processing proposed that the activation of self-representations can modulate the mapping between stimuli and perception, memory, and decision making, and also between different stages of information processing (Sui and Humphreys, 2015).

Another possible explanation is that the ability to distinguish one from others is undeniably central to self-consciousness (David et al., 2006). Given that the fact that the self-referential processing enables children with dyslexia to take the perspective of themselves for self-related information and they have a clear self-consciousness. Thus, when they processed the self-related information, they dealt with “self” in the process of “myself” information, and when they processed the others-related information, they dealt with “others” in the process of “him/herself” information. The mean RT reflected differences in this process of recognition of the self and others, as the mean RT was increased and percent correctness scores were decreased in other-perspective condition as opposed to the self-perspective condition (Vogeley et al., 2004; D’Argembeau et al., 2007). These reasons may explain why self-referential processing showed facilitation effect of early stage of visual spatial attention for children with dyslexia. We thus demonstrated that associating neutral geometric shapes with the self has an important impact on visual spatial attention of children with dyslexia.

With cue effect as the index, the present study also indicated that the regulatory role of self-referential processing was significantly larger than other-referential processing in early visual spatial attention orientation stage. This phenomenon showed a high degree of consistency between children with dyslexia and typically developing readers. Thus, we concluded that the self-referential processing might play an important modulatory role in early stage of visual spatial attention in children with dyslexia. The findings of the present study reveal the cognitive processing mechanism of the self-referential processing in visual spatial attention deficit of children with dyslexia and typically developing readers. Self-related information is a cue that has special biological significance to individuals. Previous research showed that self-related information, as socially salient information, showed faster processing speed, better memory performance, and automatically captures attention and is hard to ignore (Brédart et al., 2006). Moreover, self-related information can also be preferentially identified in the condition of subliminal priming. When primes displayed for 33 ms (peri-liminal), even primes displayed for 17 ms, there was a significant difference only in reaction time of self-target faces, compared to the nonself-target faces (Pannese and Hirsch, 2010). Compared to other-related information, the self-related information also reflected the automatic, unintentional, unconscious, and uncontrolled aspects of attention capture (Alexopoulos et al., 2012). Therefore, the self-related information can also automatically capture the attention resources in a bottom-up fashion when the cue-target interval is very short. In this condition, attention resources can be shifted and oriented quickly and preferentially to self-related information, resulting that children with dyslexia promote the effective shift in the early stage of visual spatial attention during self-referential processing and enhance their response speed to the valid cue position of target stimuli.

The current investigation has some limitations that might be addressed in future research. First of all, the participants in the present study were only the third-grade to fifth-grade students, which may limit the generalizability of the findings. Future research should explore whether the self-referential processing modulating the early stage of visual spatial attention is maintained in younger students. Second, future investigations should consider the regulation effect of the self-referential processing on the inhibition of return of visual spatial attention for children with dyslexia. On the basis of previous studies, we divided the information processing of visual spatial attention into two stages: (1) the early stage of attentional processing (about 100 ms) – in this stage, attention can shift for the first time; and (2) the stage of inhibition of return effect during later attentional processing (after 300 ms) (Posner, 1984; Facoetti et al., 2000, 2003a,b,c; Ruffino et al., 2014). Accordingly, we also have observed the cue effect of self-referential processing was significantly higher than the other-referential processing only in the early stage of visual spatial attention shifting, while the cue effect was not significant in the later stage of inhibition of return of visual spatial attention. Recently, a study indicated that, in shorter SOAs, because of the lower preparation function of cues in the visual spatial attention, which lead to the Foreperiod effect, different SOAs were an important factor influencing the inhibition of return effect in visual spatial attention (Silvert and Funes, 2016). Therefore, the regulation effect of the self-referential processing on the inhibition of return of visual spatial attention for children with dyslexia is a problem that needs further study in the future. The present study provides a feasible paradigm for the research into the inhibition of return of visual spatial attention in children with dyslexia.

The findings of the present study have some important contributions for research. We manipulated the reference task systematically in the cue-target paradigm and investigated the modulation effect of self-referential processing on visual spatial attention of children with dyslexia. In the self-referential processing condition, we showed that children with dyslexia demonstrated stable cue effects in the visual spatial attention orientation tasks when the SOAs were set to 100 ms, while other-referential processing weakened the cue effects of the visual spatial attention orientation of children with dyslexia. Our analyses suggest that self-referential processing can regulate the visual spatial attention deficits of children with dyslexia. This finding broadens the view about the role of self-reference processing in the link between self-related information and visual spatial attention of children with dyslexia.

In conclusion, the results of the present study indicated that self-referential processing regulated the early stage of visual spatial attention processing in children with dyslexia, while the other-referential processing weakened the cue effect of visual spatial attention orientation. With the cue effect as an indicator, the findings also revealed that the regulatory effect of self-referential processing further facilitated in terms of variations in the efficiency of the early stage of attentional shift.

## Data Availability Statement

All datasets generated for this study are included in the manuscript/supplementary files.

## Ethics Statement

This study was carried out in accordance with the recommendations of Psychological and Behavioral Research Ethics Committee of The Northwest Normal University, with written informed consent obtained from the parent or teacher of each participant. The protocol was approved by the Psychological and Behavioral Research Ethics Committee of The Northwest Normal University.

## Author Contributions

AZ and BD contributed to the study design. BD, MW, WW, ML, XM, and YT collected the data. AZ and BD performed data analyses and drafted manuscript. All authors provided critical revisions on the manuscript draft and approved the final version of the manuscript.

### Conflict of Interest

The authors declare that the research was conducted in the absence of any commercial or financial relationships that could be construed as a potential conflict of interest.
